# Differentiation of Perilesional Edema in Glioblastomas and Brain Metastases: Comparison of Diffusion Tensor Imaging, Neurite Orientation Dispersion and Density Imaging and Diffusion Microstructure Imaging

**DOI:** 10.3390/cancers15010129

**Published:** 2022-12-26

**Authors:** Urs Würtemberger, Alexander Rau, Marco Reisert, Elias Kellner, Martin Diebold, Daniel Erny, Peter C. Reinacher, Jonas A. Hosp, Marc Hohenhaus, Horst Urbach, Theo Demerath

**Affiliations:** 1 Department of Neuroradiology, Medical Center—University of Freiburg, Faculty of Medicine, University of Freiburg, 79106 Freiburg, Germany; 2 Department of Diagnostic and Interventional Radiology, Medical Center—University of Freiburg, Faculty of Medicine, University of Freiburg, 79106 Freiburg, Germany; 3 Department of Stereotactic and Functional Neurosurgery, Medical Center—University of Freiburg, Faculty of Medicine, University of Freiburg, 79106 Freiburg, Germany; 4 Department of Medical Physics, Medical Center—University of Freiburg, Faculty of Medicine, University of Freiburg, 79106 Freiburg, Germany; 5 Institute of Neuropathology, Medical Center—University of Freiburg, Faculty of Medicine, University of Freiburg, 79106 Freiburg, Germany; 6 IMM-PACT Clinician Scientist Program, Faculty of Medicine, University of Freiburg, 79106 Freiburg, Germany; 7 Berta-Ottenstein-Program for Advanced Clinician Scientists, Faculty of Medicine, University of Freiburg, 79106 Freiburg, Germany; 8 Fraunhofer Institute for Laser Technology, 52074 Aachen, Germany; 9 Department of Neurology and Neurophysiology, Medical Center—University of Freiburg, Faculty of Medicine, University of Freiburg, 79106 Freiburg, Germany; 10 Department of Neurosurgery, Medical Center—University of Freiburg, Faculty of Medicine, University of Freiburg, 79106 Freiburg, Germany

**Keywords:** glioblastoma, brain metastasis, diffusion magnetic resonance imaging, diffusion tensor imaging, neurite orientation dispersion and density imaging, diffusion microstructure imaging, multicompartment model, peritumoral edema

## Abstract

**Simple Summary:**

Perilesional T2 hyperintensity in glioblastomas and brain metastases shows neuropathologically detectable differences in the extent of edema formation. We compared novel diffusion microstructure imaging (DMI) with the more established NODDI and DTI techniques to determine if they could detect differences in free water content. Using DMI V-CSF and DTI MD, we found significant differences between glioblastomas and brain metastases in this regard but not with NODDI V-ISO.

**Abstract:**

Although the free water content within the perilesional T2 hyperintense region should differ between glioblastomas (GBM) and brain metastases based on histological differences, the application of classical MR diffusion models has led to inconsistent results regarding the differentiation between these two entities. Whereas diffusion tensor imaging (DTI) considers the voxel as a single compartment, multicompartment approaches such as neurite orientation dispersion and density imaging (NODDI) or the recently introduced diffusion microstructure imaging (DMI) allow for the calculation of the relative proportions of intra- and extra-axonal and also free water compartments in brain tissue. We investigate the potential of water-sensitive DTI, NODDI and DMI metrics to detect differences in free water content of the perilesional T2 hyperintense area between histopathologically confirmed GBM and brain metastases. Respective diffusion metrics most susceptible to alterations in the free water content (MD, V-ISO, V-CSF) were extracted from T2 hyperintense perilesional areas, normalized and compared in 24 patients with GBM and 25 with brain metastases. DTI MD was significantly increased in metastases (p = 0.006) compared to GBM, which was corroborated by an increased DMI V-CSF (p = 0.001), while the NODDI-derived ISO-VF showed only trend level increase in metastases not reaching significance (p = 0.060). In conclusion, diffusion MRI metrics are able to detect subtle differences in the free water content of perilesional T2 hyperintense areas in GBM and metastases, whereas DMI seems to be superior to DTI and NODDI.

## 1. Introduction

In the most common adult intracranial malignancies, glioblastomas (GBM; IDH wild type) and brain metastases, the central tumor is usually surrounded by T2 signal elevation in the perilesional brain tissue. Histopathologically, in GBM this area represents an infiltrative edema with evidence of tumor cells [1,2], whereas in brain metastases it reflects a primarily vasogenic edema [3]. Both tumor types may present with morphologically comparable MR imaging features of a central tumor mass, i.e., with marginal contrast enhancement and central necrotic areas.

Since the diagnostic workup and treatment regimen of GBM and metastases differ, early and reliable differentiation of the two entities has clinical significance [4,5]. Advanced imaging techniques might therefore help to avoid invasive procedures, which could reduce sampling errors as well as intervention-related complications. 

Diffusion-based MRI (dMRI) techniques are a valuable tool to non-invasively investigate the brain tissue´s microstructure. For this, dMRI assesses the spatial diffusion of water molecules, which, among other factors, depends on the cytoarchitecture and cell size and is thus influenced by cellular structures such as cell membranes. Increased cell density as found in cellular-rich tumors or cytotoxic edema caused by ischemia results in restricted diffusion. Advanced dMRI techniques can provide information not only on the cellularity of intracranial masses but also on their associated perilesional white matter changes.

Several studies used diffusion tensor imaging (DTI) for the analysis of perilesional white matter changes. DTI describes the movement of water molecules with parameters such as fractional anisotropy (FA) and mean diffusivity (MD), which correspond to the directionality and magnitude of water movements within a voxel, considering the voxel as a single compartment. A meta-analysis described increased FA and/or decreased MD in the perilesional region of high-grade gliomas compared to brain metastases [6], but there are other studies investigating both FA and MD of perilesional edema in glioblastoma and brain metastases with diverging results [7,8,9]. For example, one study demonstrated increased FA in brain metastases compared to grade 2 and grade 3 gliomas, but no significant differences in FA and MD were found when compared to glioblastomas [8]. In contrast, a small prospective study reported significantly increased MD and decreased FA in brain metastases compared with GBM [10].

Compared to DTI, multicompartmental approaches permit more specific insight on microstructure. For this, they rely on a standard white matter model that defines three compartments [11,12,13]: (1) the free water/CSF fraction in that molecules randomly move at the distance of their diffusion length (in the range of tenth of micrometers); (2) the volume fraction within neuronal processes (i.e. axons and dendrites) with almost one-dimensional molecule diffusion due to tight membrane borders; and (3) the volume fraction outside of axons or dendrites, characterized by an intermediate constraint to molecule diffusion representing the cellular compartment and the extracellular matrix. 

Neurite orientation and dispersion imaging (NODDI) is the most widely used technique to disentangle the contribution of these compartments to the dMRI signal and estimate volume fraction parameters based on different water diffusion behavior: isotropic free diffusion (V-ISO), restricted intracellular diffusion within axons and cells (V-IC) and hindered extracellular diffusion (V-EC). Furthermore, the orientation dispersion index (ODI) estimates the intravoxel variability of fiber orientation. These parameters are calculated by a maximum a posteriori estimator which thus relies on hard a priori constraints. Due to this, the assessment of NODDI parameters in pathologically altered tissue is hampered. To overcome this, the recently introduced diffusion microstructure imaging (DMI) technique relaxes these hard constraints by using assumed smooth and biophysically motivated prior distributions by means of a Bayesian estimator [13]. 

This approach was successfully used in studies on other neurological disorders, showing increased free water volume fraction (V-CSF) in periventricular T2 hyperintense caps in patients with normal pressure hydrocephalus compared to healthy controls [14], a reduction in intra-axonal volume (V-intra) in patients with unilateral temporal lobe epilepsy and hippocampal sclerosis could be demonstrated and confirmed by electron microscopy [15], widespread white matter edema in patients after SARS-CoV2-infection otherwise not detectable in conventional structural imaging [16] and deeper insight into the pathophysiology of neurodegenerative Parkinson syndromes [17]. In this way, DMI also proves directly useful diagnostic relevance since it can detect pathological changes even in brain areas that previously appeared normal by means of structural imaging.

Regarding brain tumors, DMI was employed to assess perilesional T2 signal alterations in GBMs and brain metastases and revealed significant differences regarding perilesional free water content with corroborating findings in neuropathology [18].

In line with these results, a significantly increased V-EC was described in the perilesional T2 signal change around glioblastomas in a small group of patients [19]. In contrast to DMI [18], NODDI-based studies failed to demonstrate a significant difference of free water fraction (V-ISO) within peritumoral edema [20], which conceptually should correspond to DMI V-CSF. 

In order to clarify this discordance, we sought to directly compare free water sensitive DTI, NODDI, and DMI metrics to investigate the perilesional tissue in GBM and brain metastases. We hypothesized that DMI is more susceptible to alterations in the perilesional free water compartment in GBM and brain metastases than DTI or NODDI. 

## 2. Materials and Methods

### 2.1. Patient Selection and Imaging

For this retrospective study, we included patients presenting with an intra-axial contrast-enhancing mass lesion scanned in a 3-year period (2019–2022). Patients with relevant small-vessel disease (Fazekas > 1), concomitant vascular lesions (e.g., vascular malformations) or imaging features of neurodegenerative disorders (e.g., Alzheimer’s disease, frontotemporal lobar degeneration, cerebral amyloid angiopathy) were excluded. Similarly, previous tumor resections and brain biopsies, prior radiation therapy, and poor image quality led to study exclusion. To account for potential steroid-related effects, subgroup-analyses were complemented by excluding patients with previous therapy with corticosteroids, since doses and temporal relation to time interval before imaging were not standardized and individually adapted.

Imaging was performed with 3 Tesla MRI scanners (MAGNETOM Prisma and MAGNETOM Prisma FIT, Siemens Healthcare, Erlangen, Germany) using a 64-channel head and neck coil including advanced diffusion MRI and isotropic T1w data pre- and post Gd-administration and isotropic FLAIR (fluid-attenuated inversion recovery) sequences for anatomical delineation and segmentation. Post-contrast T1w sequences were acquired 4–5 min after intravenous injection of 0.1 mmol/kg gadobutrol (ProHance®, Bracco Imaging, Milan, Italy).

T2-weighted (T2w) isotropic 3D FLAIR images were acquired (repetition time: 5000 ms; echo time 388 ms; flip angle: variable; TI 1800 ms; 1.0 mm isotropic voxels; 160 contiguous sagittal slices). T1-weighted (T1w) images were acquired with three-dimensional (3D) magnetization-prepared 180° radio-frequency pulses and a rapid gradient-echo (MP-RAGE) sequence (repetition time: 2500 ms; echo time: 2.82 ms; flip angle: 7°, TI = 1100 ms; GRAPPA factor = 2; 1.0 mm isotropic voxels; 192 contiguous sagittal slices). Diffusion MRI sequences were acquired with the following parameters: axial orientation, 42 slices, voxel size 1.5 × 1.5 × 3 mm³, TR 2800 ms, TE 88 ms, bandwidth 1778 Hz, flip angle 90°, simultaneous multi-band acceleration factor 2, GRAPPA factor 2, 15 non-diffusion weighted images, 2 × 58 images with b-factors 1000 and 2000 s/mm^2^; 17 diffusion directions for b-factor 0 and 57 diffusion directions each for b-factors 1000 and 2000 s/mm^2^; acquisition time was 6:22 min.

The study was conducted in accordance with the 1964 Helsinki Declaration and its later amendments and approved by the local ethics committee. Informed written consent was waived by the local ethics committee (Ethics Committee, Freiburg University Medical Center) due to the purely retrospective analysis. We hereby confirm that all methods were performed in accordance with the relevant guidelines and regulations.

### 2.2. Image Postprocessing and ROI Based Analysis

Data processing was conducted in our in-house post-processing platform NORA (www.nora-imaging.org; last accessed on 24 October 2022). T1w image datasets were automatically segmented into white matter, gray matter, and cerebrospinal fluid (CSF) with SPM12 (Wellcome Centre for Human Neuroimaging, London, UK). T2w and dMRI datasets were coregistered to the T1w images. Validity of the coregistrations was visually confirmed. 

Preprocessing of diffusion MRI data included denoising [21], Gibbs-ringing artifacts-correction [22] and upsampling to isotropic resolution of 1.5 mm^3^ [13]. 

DTI measures were obtained from b  =  0 and 1000 s/mm^2^ images using a publicly available open source toolbox (https://www.uniklinik-freiburg.de/mr-en/research-groups/diffperf/fibertools.html, accessed on 27 October 2022) using the ordinary log-linear fitting, calculating the mean diffusivity (MD). Since MD is technically most susceptible to non-directional free water diffusivity, DTI analysis was limited to this parameter. NODDI V-ISO was calculated with the accelerated microstructure imaging via convex optimization (AMICO) method, a regularized version of NODDI with faster processing times due to the linearization of fitting procedures [23]. DMI metrics based on a three-compartment diffusion model were estimated using a Bayesian approach [13,18] determining the free water fraction V-CSF.

**Figure 1:**
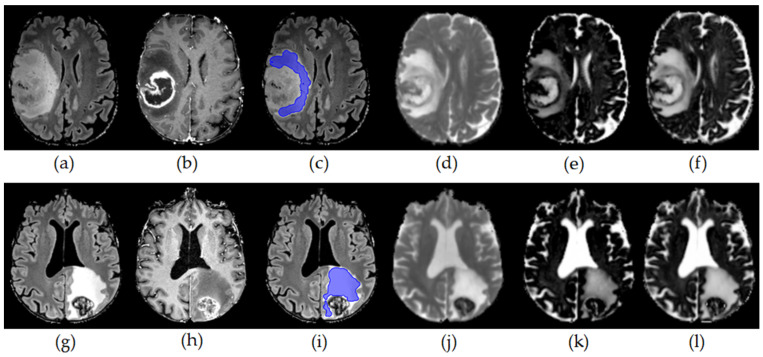
Upper row: Axial FLAIR ((**a**,**c**) with superimposed perilesional T2 area segmentation), axial T1 MPRage post-Gd (**b**) and parametric maps for MD (**d**), V-ISO (**e**) and V-CSF (**f**) in a 60-year-old female patient with a right frontoparietal glioblastoma. Lower row: Axial FLAIR ((**g**,**i**) with superimposed perilesional T2 area segmentation), axial T1 MPRage post-Gd (**h**) and parametric maps for MD (**j**), V-ISO (**k**) and V-CSF (**l**) in a 51-year-old male patient with a left parietal esophageal cancer metastasis.

**Figure 2:**
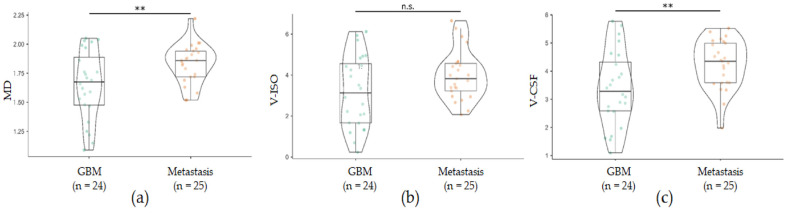
Perilesional diffusion metrics in patients with GBM (*n* = 24) and metastases (*n* = 25), normalized to whole-brain normal-appearing white matter (NAWM) values. Compared to GBM, in metastases there is a significant shift towards increased mean diffusivity ((**a**); MD) and increased interstitial free water ((**c**); V-CSF). Isotropic free diffusion ((**b**); V-ISO) shows a trend towards increased values in metastases but does not reach significance. ** *p* ≤ 0.01.

Perilesional T2w hyperintense areas were manually segmented by two neuroradiologists with 5 and 7 years of clinical neuroimaging experience on isotropic 3D FLAIR images in co-registration with 3D T1w post-Gd datasets to avoid erroneous segmentation of contrast-enhancing tumor components. To account for potential partial volume effects, we carefully excluded contrast enhancing outer tumor margins and adjacent gray matter (exemplary cases are presented in Figures 1 and 2). 

To account for age-related white matter alterations [24], we normalized the dMRI parameters to the respective normal appearing white matter (NAWM) mean value. For this, NAWM was defined as the WM after exclusion of the tumor mass and the perilesional T2w hyperintensity from the SPM12-based white matter segmentation maps (tissue probability value > 0.5). 

### 2.3. Statistical Analysis

Data distribution was tested with Shapiro–Wilk test. Patient age as well as the perilesional T2w hyperintensity volume were compared between GBM and metastases using the Mann–Whitney-U test. A One-way ANCOVA, controlling for lesion volume, was conducted between perilesional T2 areas comparing GBM and metastases groups and Bonferroni correction was employed to account for multiple comparisons. The Pearson’s correlation coefficient was used to relate T2 volumes to dMRI derived metrics MD, V-ISO, and V-CSF. We plotted the receiver operating characteristic (ROC) curves of GBM and metastasis perilesional and MD, V-ISO and V-CSF. Values with an α-level of 0.05 were considered statistically significant. All statistical analyses were performed using R statistics V. 4.0 (R Core Team 2020, Bell Laboratories, Holmdel, NJ, USA; https://www.R-project.org; last accessed 27 October 2022). Boxplots were calculated using CRAN.R-packages (https://CRAN.R-project.org/package=ggplot2, last accessed 12 December 2022, https://CRAN.R-project.org/package=ggstatsplot, last accessed 27 October 2022). The ROC curves were visualized using IBM SPSS, version 22. The Test ROC module was used to calculate ROC analyses and DeLong test, which is built on the cutpointR module version 1.1.1 (https://CRAN.R-project.org/package=cutpointr, last accessed 27 October 2022). 

## 3. Results

### 3.1. Study Population

We report on 49 patients with contrast-enhancing intracranial mass lesions and T2/FLAIR signal elevations in perifocal brain tissue that underwent presurgical MRI including multishell dMRI. Of those, histopathology confirmed an IDH wild-type GBM in 24 patients (11 female; mean age: 65.5; SD 13.1, range 41.8–88.0 years) whereas 25 patients (12 female; mean age: 65.5; SD 12.1, range 46.5–87.2 years) had brain metastases. Primary tumors in patients with brain metastases comprised lung cancer (*n* = 13), melanoma (*n* = 5), breast cancer (*n* = 2), urothelial carcinoma (*n* = 1), colorectal carcinoma (*n* = 1), esophageal carcinoma (*n* = 1), ovarian cancer (*n* = 1) and thymus carcinoma (*n* = 1). Both groups did not differ in terms of age (*p* = 0.83), sex (*p* = 0.826) or total volume of perilesional T2 hyperintensity (*p* = 0.73).

Corticosteroids had been administered in 10/24 patients in the GBM group and 9/25 patients with metastases. Due to the retrospective evaluation, the exact temporal relation of steroid administration to the time of imaging could not be determined.

### 3.2. Increased Free Water in Perilesional T2w Hyperintensities in GBM Compared with Brain Metastases 

ROI-derived diffusion metrics were normalized to the mean of whole-brain NAWM and compared between GBM and metastases groups (exemplary in Figure 1), controlling for perilesional T2 hyperintense area volume and age. There was a significant increase in MD (F (1,1) = 8.18, *p* = 0.006) and DMI V-CSF (F (1,1) = 12.03, *p* = 0.001) in brain metastases compared to GBM (Table 1, Figure 2). There was a tendency towards increased NODDI V-ISO (F (1,1) = 3.72, *p* = 0.060). 

The distribution of individual values showed that group differences were particularly driven by decreased values (MD, V-ISO, V-CSF) in part of the GBM as also evidenced by lower Min (MD, V-ISO, V-CSF) values as well as lower 25% percentiles in GBM compared to metastases (Table 1, Figure 2).

A further subgroup analysis excluding patients with prior corticosteroid treatment led to similar results with an increase in MD (F (1,1) = 8.27, *p* = 0.008) and DMI V-CSF (F (1,1) = 9.02, *p* = 0.006) and a tendency towards increased NODDI V-ISO (F (1,1) = 3.38, *p* = 0.077) in brain metastases (Table 2). 

### 3.3. Increased Free Fluid in Perilesional T2w Hyperintensities Are Associated with Perilesional Area Volume and Age

Results of the Pearson’s correlation indicated a positive association between perilesional T2 volume and MD in GBM (*r* = 0.50, *p* = 0.012). In both groups we also found a negative correlation between patient age and perilesional V-CSF (metastases: *r* = −0.72, *p* < 0.001; GBM: *r* = −0.69, *p* < 0.001) and V-ISO (metastases: *r* = −0.54, *p*= 0.009; GBM: *r* = −0.52, *p* = 0.01). In GBM, there also was a significant negative correlation between age and MD (*r* = −0.65, *p* < 0.001). 

There was a positive correlation between all three diffusion metrics within the groups, confirming that all three diffusion metrics reflect the distribution of free water: in metastases between MD and V-CSF (*r* = 0.68, *p* = 0.002), MD and V-ISO (*r* = 0.51, *p* = 0.009) and V-CSF and V-ISO (*r* = 0.82, *p* < 0.001) and in GBM between MD and V-CSF (*r* = 0.96, *p* < 0.001), MD and V-ISO (*r* = 0.85, *p* < 0.001) and V-CSF and V-ISO (*r* = 0.90, *p* < 0.001).

### 3.4. Comparative ROC Analysis of DTI MD, NODDI V-ISO and DMI V-CSF

**Figure 3:**
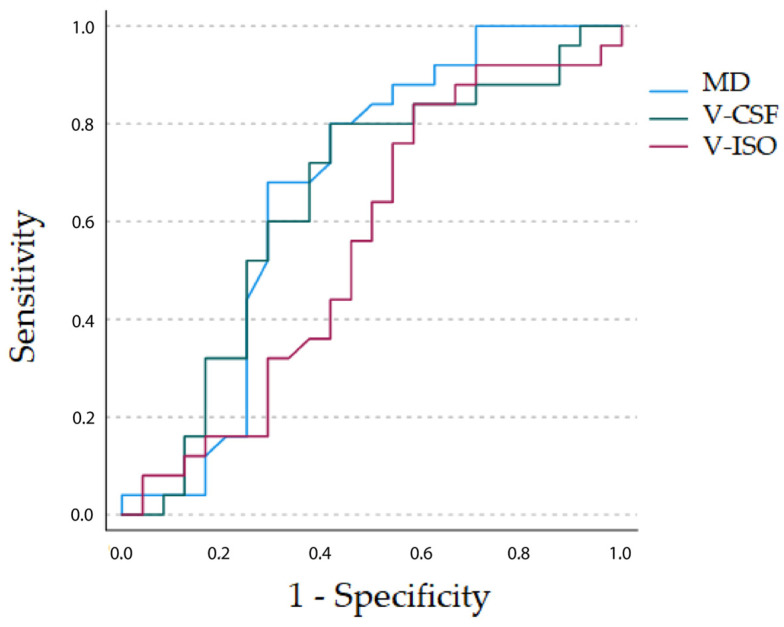
ROC curves of diffusion metrics obtained from perilesional T2 hyperintensities in 24 patients with GBM and 25 with brain metastases showing a predictive value, which is higher for perilesional MD (AUC 0.669) and V-CSF (AUC 0.700) than V-ISO (AUC 0.636) regarding the presence of a GBM.

Building on the systematic differences regarding diffusion metrics of perilesional T2 hyperintensities between GBM and metastasis groups, we conducted an ROC cut-point analysis to determine thresholds for an optimal separation for MD, V-CSF and V-ISO data. A model equally weighted for sensitivity and specificity supported the affiliation to the GBM cohort for normalized MD when applying an upper threshold of 1.72 (sensitivity, 80%; specificity, 58%) and for V-CSF when applying a threshold of 3.51 (sensitivity, 84%; specificity, 54%). There was a slightly higher sensitivity but lower specificity when using V-ISO, applying a threshold of 2.66 (sensitivity, 92%; specificity, 46%). There was no significant difference between AUCs of MD (AUC 0.669; DeLong *p* = 0.376 vs. V-CSF, *p* = 0.526 vs. V-ISO), V-CSF (AUC 0.700; DeLong *p* = 0.154 vs. V-ISO) and V-ISO (AUC 0.636). ROC curves are presented in Figure 3. 

Discrimination between GBM and metastases was further improved when only considering MD, V-ISO and V-CSF values below the 25th percentile with an AUC of 1.00 for MD_25_ and V-ISO_25_ and 0.976 for V-CSF_25_ but not for values above the 75th percentile with AUCs of 0.274 for MD_75_, 0.457 for V-ISO_75_ and 0.500 for V-CSF_75_.

## 4. Discussion

In this study, we compared the potential of different dMRI models to determine the proportion of free water within the perilesional T2 hyperintensity in pathologically confirmed GBM and brain metastasis patients as a possible discriminatory parameter.

Thus, building on our previous study [18], we were able to demonstrate an increased free water compartment in brain metastases compared to GBM patients in a now larger patient population, where V-CSF seems to the strongest parameter, followed by DTI-based MD. In contrast, NODDI-based V-ISO, although conceptually most comparable to DMI-based V-CSF, did not significantly differ between both entities, even though there was a tendency for increased perilesional V-ISO in metastases.

In addition, correlation analysis revealed within both groups a relationship between patient age and perilesional V-CSF and V-ISO and, in GBM, also to mean diffusivity, and thus age was statistically included as a covariate. As expected, there was a strong positive correlation between all three diffusion metrics, which is well compatible with an increase of free water in the interstitial space that can be detected by various metrics, including conventional ones such as MD.

Our finding of significantly increased conventional DTI-based MD in brain metastases is consistent with several studies that employed DTI in the perilesional region to distinguish high-grade gliomas and/or GBM from metastases [10,25,26]. However, it should be emphasized that one study in comparable population size also showed non-significant differences in this regard [20]. Taken together, these results indicate a higher non-directional water diffusivity in metastases, reflecting pure vasogenic edema. As DTI techniques refer to a single-compartment-type model, MD may be affected by both edematous changes in the context of a disrupted blood–brain barrier and microstructural tissue damage such as axonal degradation with consecutive demise of white matter fiber organization. This can be explained by the fact that in conventional DTI (without using the technique of “free water elimination”), free water is not considered as independent [27]. In contrast, biophysically motivated multicompartimental approaches such as NODDI or DMI offer a more specific insight on the distribution of microstructural compartments [11,13]. By additional investigation of the multicompartment metrics, we were able to assign the MD elevation to an increased free water fraction.

Although NODDI is the most widely applied multicompartmental dMRI model to date [28], no significant differences in the extent of free water were found in contrast to DMI-derived V-CSF. In line with this, the direct comparison of these two multicompartment models in our study proves the better suitability of DMI and especially V-CSF for the characterization of perilesional tissue changes in white matter, confirming previous histopathological findings [18]. As mentioned earlier, the faster post-processing time of the DMI approach favors its use in standard preoperative MRI measurements. Acceptance for the use of novel MR applications depends not only on the time required for the examination but also on the amount of post-processing required. In order to create a roughly comparable time requirement between DMI and NODDI here as well, we applied the AMICO [23] technique to NODDI, which enables a massive acceleration of the NODDI analysis reducing processing times of several orders of magnitude. In this context, we do not expect any relevant impact on the free water estimation, as AMICO NODDI should even allow slightly more accurate and robust parameter estimates than the nonlinear NODDI approach.

Although an antiedematous effect of corticosteroid application in GBM and brain metastases has been reported in the literature [29], we did not detect a significant effect in our results, which is consistent with previous findings [18].

Preoperative tumor classification and thus differentiation of glioblastoma from solitary brain metastases could have implications for current diagnostic planning: In GBM, primary resection of at least the contrast-enhancing tumor portion is aspired as a both diagnostic and therapeutic procedure, whereas in brain metastases, a search for additional lesions, for example, by whole-body tumor staging is often performed prior resection [30]. Diagnostic confirmation is in metastases often oriented to the most easily accessible, often extracranial lesion. As far as the neurosurgical approach is concerned in GBM—in contrast to generally well circumscribed brain metastases—an extended resection beyond the contrast-enhancing tumor area if possible, including perilesional FLAIR alterations [31], is associated with improved survival [32]. This can be explained by the fact that GBM shows tumor infiltration within the perilesional edema and beyond [33,34] and recurrence usually occurs adjacent to the resection area [35]. The edema surrounding brain metastases is usually vasogenic without infiltrating zone, which may also be underlined by the finding that in metastases, in contrast to GBM, nearly homogeneous ADC values were found both in the peritumoral edema directly surrounding the contrast-enhancing tumor and in the edema closest to the normal-appearing white matter [36]. 

As our results yet do not support accurate differentiation in this regard, an additional multiparametric MR approach to better characterize the perilesional tumor area, e.g., with additional diffusion, perfusion- or spectroscopy-derived metrics, seems obvious and may allow more accurate differentiation [37]. The wider spread of values (MD, V-CSF, V-ISO) in GBM compared to metastases suggests that peritumoral T2 hyperintensity in these tumors may be subject to inter- and possibly also intraindividual heterogeneity with respect to the distribution of free water. This is reflected in the higher SD in GBM as well as also a remarkable shift to lower 25% percentiles, which could, in principle, have diagnostic value as shown in the ROC analysis.

The direct comparison of these two multicompartment models is a major strength of our study, as in conjunction with our previous work, our results undermine the better suitability of DMI and especially V-CSF for the characterization of perilesional free water. Another relevant strength of our study is the higher number of cases and also the well-matched and characterized tumor groups for which there were no significant differences in terms of sex, age, perilesional FLAIR volume and proportion of dexamethasone doses received prior to imaging. 

As a limitation, we did not include further, multimodal MRI data in our analyses, which should be aimed for in the near future. Since the metastasis group contained different primary tumor histopathologies, mostly lung tumors, no definitive conclusion can be drawn regarding dependence of diffusion metrics on different tumor cell types. This concerns not least the genetic heterogeneity in GBM, which should also be the target of future analyses. 

## 5. Conclusions

The perilesional tumor area shows higher free water content in brain metastases compared to GBM. In this comparative study, we demonstrated that in contrast to NODDI V-ISO these changes are significantly measurable with DMI V-CSF and DTI-MD and that a diagnostic discrimination of GBM vs. metastasis based on them seems superior with the first two models.

## Figures and Tables

**Table 1:** Patient characteristics and normalized ROI (perilesional T2 hyperintense area)-derived diffusion metrics.

	GBM	Metastasis	*p*-Value
*n*	24	25	
Sex (m/f)	13/11	13/12	*p* = 0.826
Age (years) (SD)	65.5 (13.1)	65.5 (12.1)	*p* = 0.995
Perifocal T2 volume (ml) [IQR]	19.1 [24.4]	23.0 [41.5]	*p* = 0.729
Previous corticosteroid therapy	10/24 (41.7%)	9/25 (36.0%)	*p* = 0.696
MD [IQR]	1.67 [4.10]	1.86 [2.20]	*p* = 0.006
Min, Max 25%, 75%	1.09, 2.05 1.48, 1.89	1.52, 2.22 1.72, 1.94	
V-ISO [IQR]	3.13 [2.89]	3.82 [1.34]	*p* = 0.060
Min, Max 25%, 75%	0.235, 6.12 1.67, 4.55	2.07, 6.65 3.23, 4.57	
V-CSF [IQR]	3.29 [1.74]	4.35 [1.40]	*p* = 0.001
Min, Max 25%, 75%	1.1, 5.77 2.58, 4.32	1.98, 5.52 3.59, 4.99	

Values are given in mean and standard deviation (SD) or median and interquartile ranges [IQR].

**Table 2:** Patient characteristics and normalized ROI (perilesional T2 hyperintense area)-derived diffusion metrics, excluding patients with prior corticosteroid therapy.

	GBM	Metastasis	*p*-Value
*n*	14	16	
Sex (m/f)	7/7	7/9	*p* = 0.822
Age (years) (SD)	63.1 (12.5)	66.3 (12.1)	*p* = 0.786
Perifocal T2 volume (ml) [IQR]	19.6 [23.4]	5.1 [36.7]	*p* = 0.729
MD [IQR]	1.64 [0.48]	1.88 [0.18]	*p* = 0.008
V-ISO [IQR]	3.13 [3.32]	3.77 [1.55]	*p* = 0.077
V-CSF [IQR]	3.24 [1.99]	4.33 [1.36]	*p* = 0.006

Values are given in mean and standard deviation (SD) or median and interquartile ranges [IQR].

## Data Availability

The anonymized data presented in this study are available on reasonable request from the corresponding author.
